# Decoding adult murine pancreatic islet cell diversity through cell type-resolved proteomics and phosphoproteomics

**DOI:** 10.1038/s42003-025-08918-8

**Published:** 2025-10-17

**Authors:** Marvin Thielert, Adrian Villalba, Vincenth Brennsteiner, Maria Wahle, Constantin Ammar, Andreas-David Brunner, Alexis Fouque, Chloé Lourenço, Masaya Oshima, Latif Rachdi, Willem Staels, Matthias Mann, Raphaël Scharfmann

**Affiliations:** 1 Proteomics and Signal Transduction, Max Planck Institute of Biochemistry, Martinsried, Germany; 2 Institut Cochin, CNRS, Inserm, Université Paris Cité, Paris, France; 3 Fundación IVI, Instituto de Investigación Sanitaria La Fe, Valencia, Spain; 4 GIBIO- Bioethics Research Group, Health Department, International University of Valencia, Valencia, Spain; 5 Boehringer Ingelheim Pharma GmbH & Co. KG, Drug Discovery Sciences, Biberach an der Riss, Germany; 6 Genetics, Reproduction, and Development (GRAD), Beta Cell Neogenesis (BENE) Research Unit, Vrije Universiteit Brussel (VUB), Brussels, Belgium; 7 Division of Pediatric Endocrinology, Department of Pediatrics, Universiteit Ziekenhuis Brussel (UZ Brussel), Vrije Universiteit Brussel (VUB), Brussels, Belgium; 8 Novo Nordisk Foundation Center for Protein Research, Faculty of Health and Medical Sciences, University of Copenhagen, Copenhagen, Denmark

**Keywords:** Cell biology, Biochemistry

## Abstract

Islets of Langerhans are micro-organs scattered throughout the pancreas. They are composed of insulin-producing beta cells, glucagon-producing alpha cells, and somatostatin-producing delta cells. While their transcriptome has been extensively analyzed, protein-level information remains limited due to cell scarcity and purification challenges. Here, we combine cell sorting with highly sensitive mass spectrometry to create the first in-depth proteomic resource of pancreatic islet cells. We achieved a depth exceeding 6000 proteins per endocrine cell population, discovering new cell type-enriched ones. Deep proteomics profiling demonstrated that all three endocrine cell types were inflamed upon interferon gamma (IFNγ) treatment, a mediator of autoimmune damage in type 1 diabetes. Resolving the phosphoproteomic landscape of alpha, beta and delta cells with more than 7000 unique phosphosites per cell type provided insights into cell-specific signaling. This omics dataset offers a valuable resource for understanding pancreatic islet biology in health and disease.

## Abstract (web summary)

Here, the authors employ cell sorting and sensitive mass spectrometry to generate a comprehensive proteomic and phosphoproteomic resource, revealing inflammation responses and cell-specific signaling, advancing our understanding of islet biology in health and disease.

## Introduction

Diabetes is a group of metabolic disorders characterized by chronic hyperglycemia and caused by the destruction or dysfunction of insulin-producing beta cells. These endocrine cells are clustered in the pancreas in small micro-organs, named islets of Langerhans^1^. Adult mouse islets are composed of 75% insulin-producing beta cells, 15% glucagon-producing alpha cells and 10% somatostatin-producing delta cells^2,3^. The transcriptomic landscape of islet cells has now been extensively characterized at the single-cell level^4–6^, resulting in atlases identifying cell-specific signatures for each pancreatic endocrine cell type under basal and disease conditions^7^. Inter-species comparisons have also been explored at the transcriptome level^8^.

The dynamic proteome is thought to be a very informative reflection of the biological function of cells^9^. However, our understanding of the proteomic profiles of alpha, beta and delta cells remains limited^10,11^. Previous proteomic analyses have focused primarily on entire pancreatic islets, with limited attempts to separate the different endocrine populations. This is due to the difficulties in purifying these distinct cell populations and the limitations of mass spectrometer (MS)-based proteomics, which has struggled with the sensitivity required to profile, especially the less abundant alpha and delta cells^12,13^.

To address these challenges, we here build upon a strategy to isolate highly enriched populations of alpha, beta, and delta cells using a combination of surface markers^14^ and combine this sorting approach with recent developments in deep proteomic profiling. We employ high-sensitivity MS of trapped-ion mobility time of flight (timsTOF) instruments that allow the acquisition of proteomes from low input material, making unbiased characterization on the protein level possible^15^. Furthermore, the combination with data-independent acquisition (DIA) strategies enhances data completeness, ensuring that proteome measurements are less prone to stochastic sampling of high abundant proteins like hormones^16^.

Altogether, this approach has enabled us to build the first proteomic dataset of mouse adult alpha, beta, and delta cells. It consists of over 6000 protein groups in each endocrine cell type, an unprecedented depth of proteomic data. Additionally, our approach facilitated the detection of dynamic proteomic changes, such as the fingerprint of IFNγ, enabling exploration of proteomic alterations under inflammatory conditions. These detailed datasets provide a valuable resource for advancing research into the molecular mechanisms underlying islet biology and related diseases^17,18^. Finally, we extended our analysis to the phosphoproteomic level, mapping over 7000 unique phosphosites per cell type and elucidating cell type-specific phosphorylation patterns.

## Results

### Mouse alpha, beta, and delta cells can be resolved through FACS and proteomic profiling

**Fig. 1:**
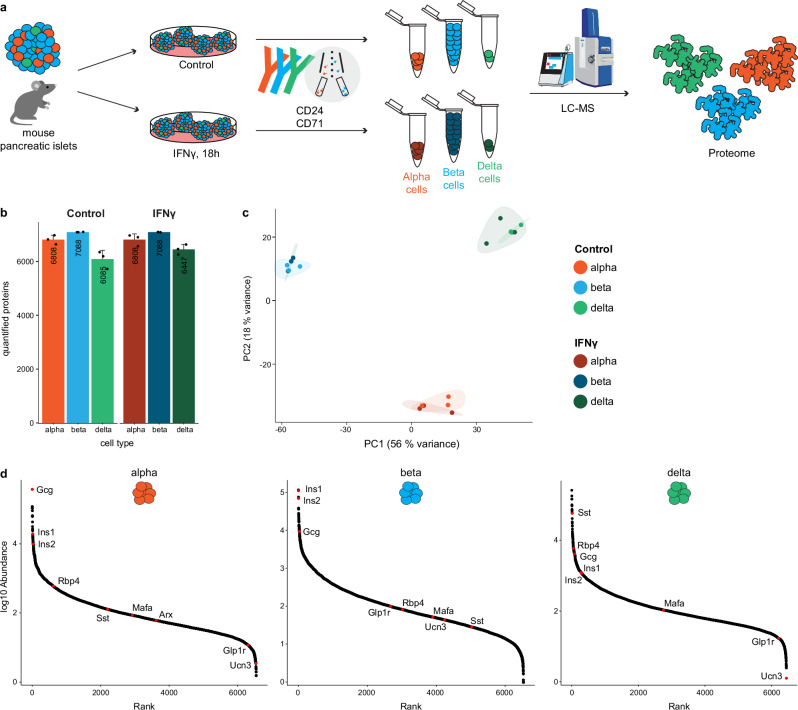
Cell type-resolved proteomic profiling of sorted male mouse adult pancreatic endocrine cells. **a** Schematic representation outlining the experimental workflow. **b** Quantification of the total number of proteins per cell type in control and IFNγ-treated conditions. Each point corresponds to a pool of islets harvested from 3 different mice. Error bars represent the standard error of the mean (SEM) calculated from *n* = 3 biological replicates. **c** PCA analysis of the complete proteomic dataset showing PC1 vs PC2, highlighting the major variation between cell types. **d** Log-rank graphs depicting the total protein quantification in each endocrine cell type, including bona fide markers for each cell type.

While global proteomic profiling has previously been described even for single whole mouse pancreatic islets^19^, these analyses were dominated by beta cell proteins due to their abundance when compared to the other islet cell types. In contrast, individual proteomic profiles of alpha, beta, and delta cell subpopulations have remained elusive. To address this gap, we prepared islets from 12-week-old C57BL/6 mice, cultured them overnight and dispersed them into single cells. We then employed our previously described FACS-based approach on alpha, beta, and delta cell populations^14^ to flow sort with antibodies against CD24 and CD71 in the Epcam^+^ epithelial fraction after excluding non-pancreatic lineages and dead cells (CD31^+^, CD45^+^, CD235a^+^ and propidium iodide^+^) (Fig. 1a and Supplementary Data 1, Supplementary Fig. 1 and Supplementary Data 2). To verify the purity of our sorted cell populations, we analyzed the mRNA expression of endocrine, acinar, and ductal markers by RT-qPCR. As expected, sorted alpha, beta, and delta cell subsets were consistently enriched in *Gcg*, *Ins1*, and *Sst*, respectively (Supplementary Fig. 2 and Supplementary Data 2). Furthermore, acinar (*Amy* and *Cpa1*) and ductal (*Spp1*) markers were quasi absent in all three endocrine cell subsets (Supplementary Fig. 2). Following cell sorting, we performed proteomics on the isolated alpha, beta and delta cell populations (Fig. 1a). Starting from only 25,000 alpha and beta cells, and 14,500 delta cells, we detected more than 6000 proteins per endocrine subset (6471 ± 42, 6493 ± 3, and 6283 ± 106 protein groups, for alpha, beta, and delta cells, respectively) (Fig. 1b). Principal component analysis (PCA) revealed that alpha, beta and delta cell populations form distinct clusters (Fig. 1c). To further validate our approach, we analyzed the abundance of bona fide markers for each endocrine cell type. As anticipated, sorted alpha cell subsets were consistently enriched in Gcg and in the transcription factor Arx, beta cells were enriched in Ins1, Ins2, Glp1r and Ucn3 compared to alpha and delta cells, and delta cells were enriched in Sst and Rbp4 (Fig. 1d). As expected, Entpd3 was detected in beta cells (Supplementary Fig. 3a and Supplementary Data 2). However, it lacks absolute specificity as it is also found in delta cells, consistent with previous reports^20,21^ and the Tabula Muris dataset. Also note that by proteomics, the sorting markers Tfrc (CD71) and Itga6 (CD49f) are enriched in beta compared to alpha cell population (Supplementary Fig. 3b). We were unable to detect CD24 in our proteomic dataset due to the absence of detectable tryptic peptides in CD24 protein sequence, which prevents its identification using standard trypsin-based mass spectrometry workflows.

We extended our analysis to investigate the effects of inflammation by performing similar proteomic analyses on alpha, beta and delta cell populations sorted from islets treated in vitro with IFNγ, which mimics inflammation observed in type 1 diabetes. We detected similar numbers of proteins in all three subsets (6488 ± 35, 6488 ± 9, and 6396 ± 47 protein groups, for alpha, beta, and delta cells, respectively) and treated samples cluster close to their respective cell type (Fig. 1b, c).

We conclude that our combined strategy of FACS and proteome profiling allows unbiased quantitative analysis of the proteome of alpha, beta, and delta cell populations in control conditions and upon IFNγ treatment, providing a powerful foundation to investigate islet cell biology.

### Unraveling cell type-resolved proteome profiles for mouse alpha, beta and delta cells

**Fig. 2:**
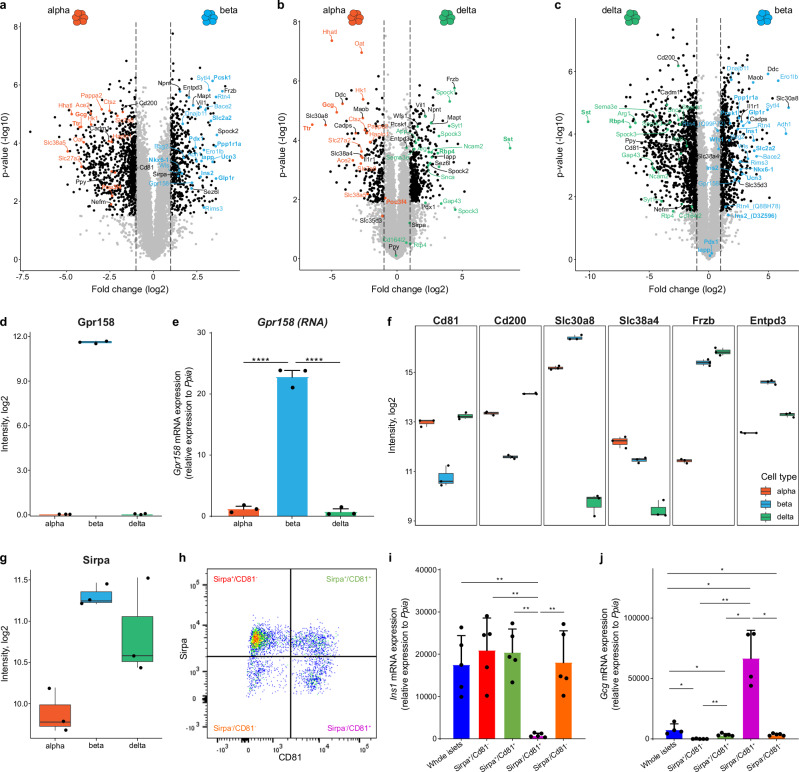
Proteomic profile of mouse alpha, beta and delta cells. **a**–**c** Volcano plots showing the differential enrichment of proteins in alpha vs beta vs delta cells. Significantly regulated proteins (adj. *p*-value < 0.05, |log2 fold change| > 1) are highlighted as black dots. Colored proteins are based on their regulation: alpha- (red), beta- (blue) and delta-specific (green), while black labels are presented in two cell types. **d** Protein levels of Gpr158 protein in alpha, beta and delta cells from proteomic measurement. Each point represents a biological sample (*n* = 3) from three independent biological replicates per cell type. **e** Relative mRNA expression levels of *Gpr158* in alpha, beta and delta cells. Biological replicates *n* = 3 per group. **f** Boxplots presenting the abundance of distinct proteins enriched in alpha, beta and delta cells. **g** Boxplot presenting the levels of Sirpa protein in alpha, beta and delta cells. Biological replicates *n* = 3 per group. **h** Representative flow cytometry plot of dispersed mouse islets. CD81 and Sirpa expression was analyzed on CD24^−^ cells after excluding dead cells (propidium iodide^+^ cells) and hematopoietic and endothelial cells (CD45-, TER119-, or CD31-positive cells). **i**, **j** Relative mRNA expression levels of endocrine cell markers *Ins1* (i) and *Gcg* (j) in whole islets and Sirpa^+^CD81^−^, Sirpa^+^CD81^+^, Sirpa^−^CD81^+^, Sirpa^−^CD81^−^ cells. *n* = 4–5 independent experiments. The symbol * represents a *p*-value less than 0.05, while ** represents a *p*-value less than 0.01.

To identify proteins enriched in alpha, beta, or delta cells, we conducted comparative analyses between these cell types (Fig. 2a–c and Supplementary Data 1). Alpha cells were enriched in established markers like Gcg, Ttr and Pou3f4 (Fig. 2a, b) and beta cells in Ins2, Nkx6.1, Pdx1, Iapp, Wfs1, Ucn3, Glp1r, Slc2a2, Pppr1a, and Pcsk1 (Fig. 2a, c). Interestingly, Pdx1, Iapp, Wfs1 and Pcsk1 were also enriched in delta cells compared to alpha cells (Fig. 2b). As expected, delta cells were specifically enriched in Sst and Rbp4, further validating our approach (Fig. 2b, c).

Our analysis revealed cell type-specific enrichment of several proteins that are either underexplored or not yet reported. Alpha cells were enriched in transporter proteins, including the fatty acid transporter Slc27a2 and the neutral amino acid transporters Slc7a8 and Slc38a5. They also exhibited higher levels of membrane proteins such as Ace2 (a carboxypeptidase involved in the cleavage of angiotensin and—interestingly—being a co-receptor for the COVID-19 virus), and Hapln1 (an extracellular matrix component) as well as enzymes including Hk1 (hexokinase 1), Ctsz (a protease), Oat (an aminotransferase), Hhatl (an acetyltransferase), and Pappa2 (a metalloprotease involved in the cleavage of Igfbp5) (Fig. 2a, b).

Beta cells demonstrated enrichment in membrane proteins like Emilin1 (involved in cell-cell contacts), Rtn4, Rims3 and Sytl4 (three regulators of vesicle exocytosis), Wls (a Wnt receptor), Bace2 (an enzyme that cleaves APP). They also showed higher levels of Adh1 (an alcohol dehydrogenase enzyme), Bag2, Dnajb11 (chaperone proteins), and Ero1lb (an enzyme involved in protein folding) (Fig. 2a, c). Notably, Gpr158, a recently deorphanized receptor^22^, was exclusively detected in the beta cell fraction (Fig. 2a, c, d). We further validated this finding by RT-qPCR, confirming *Gpr158* mRNA expression only in beta cells (Fig. 2e).

Delta cells exhibited enrichment in Gap43 and Ncam2 (membrane proteins regulating cell-cell contacts), Spock1, Spock3, and Sema3e (cell-extracellular matrix interactions), Cd164l2 and App (2 membrane proteins), Arg1 (an enzyme involved in arginine hydrolysis), Syt1, Snca, and Rtp4 (that regulate vesicle exocytosis and membrane trafficking) (Fig. 2b, c).

We also identified under-explored proteins enriched in two of the three cell types. Alpha and delta cell subsets were enriched in Cadm1 (a cell adhesion molecule), CD81 and CD200 (2 cell surface molecules), and Nefm (a cytoskeleton component), though we cannot fully exclude limited contamination by pancreatic polypeptide (PP) cells in these two populations (Fig. 2a, c, f). Alpha and beta cell subsets were enriched in Slc30a8 (a zinc transporter), Slc38a4 (an amino acid transporter), Slc35d3 (a nucleotide-sugar transporter), Cadps (a regulator of vesicle exocytosis), Ddc (DOPA decarboxylase), Maob (an oxidase located in the mitochondrial membrane), and Il1r1 (interleukin receptor) (Fig. 2b, c, f). Beta and delta cells showed enrichment for proteins such as Frzb (a Wnt-binding protein), Sez6l (a membrane receptor), Entpd3 (a nucleotidase), Vil1 (an actin-binding protein), Spock2 (a regulator of cell-cell contacts), Npnt (a ligand of integrins) and Mapt (cytoskeleton components) (Fig. 2a, b, f).

Of particular interest was Sirpa (signal regulatory protein A), enriched in both beta and delta cells (Fig. 2a, c, g). This cell surface protein has been suggested as a regulator of beta cell viability^23^. To further investigate Sirpa’s expression, we analyzed dispersed islet cells from 12-week-old mice using flow cytometry with antibodies directed against CD24, CD81 and Sirpa. After excluding the CD24^high^ population that contains delta cells^14^, we identified four subsets based on CD81 and Sirpa expression (Fig. 2h). An RT-qPCR analysis revealed the expression of *Gcg* and *Ins* in Sirpa^+^ subsets (Fig. 2i) and *Gcg* in a Sirpa^−^ fraction (Sirpa^−^/CD81^+^, Fig. 2j), supporting our proteomic analysis. Interestingly, we also observed a small subset of CD81^−^Sirpa^−^ cells (Fig. 2h) expressing *Ins* (Fig. 2i) but not *Gcg* (Fig. 2j), indicating that the majority of the beta cells express Sirpa on their surface, while a minor beta cell subpopulation does not.

### IFNγ treatment induces distinct proteomic changes in pancreatic endocrine cell populations

**Fig. 3:**
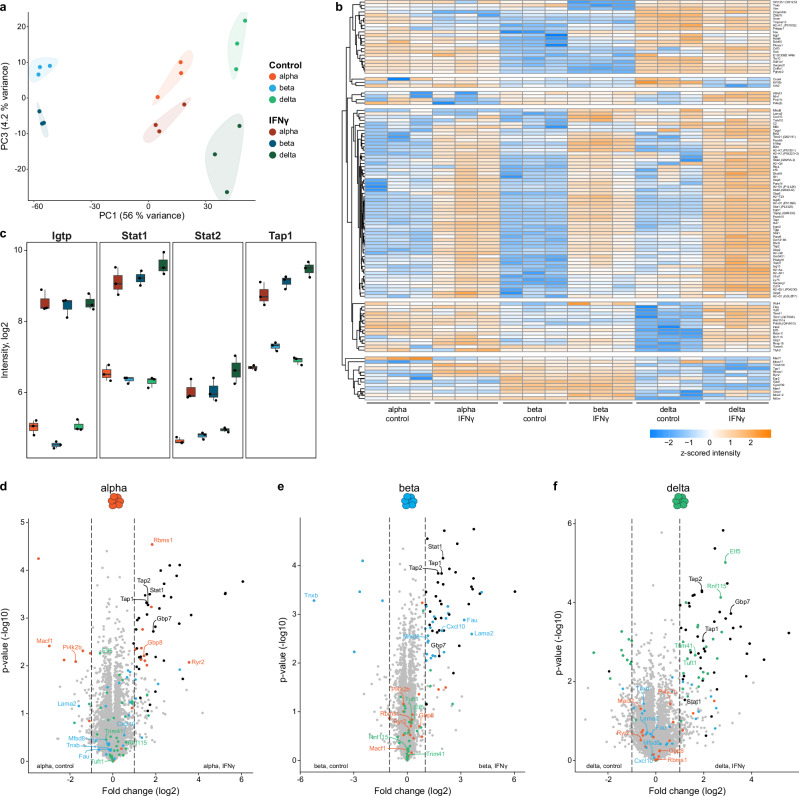
Proteomic profile of alpha, beta and delta cells upon IFNγ exposure. **a** PCA analysis of the same proteomic dataset as in Fig. 1c, showing PC1 vs PC3 to emphasize IFNγ treatment effects (PC3, 4% variation) while maintaining cell type context (PC1). **b** Heatmap comparing the proteomic profiles of control and IFNγ-treated conditions in alpha, beta and delta cells. Enriched proteins are clustered into six categories by k-means clustering. Each cluster reveals cell type-specific (cluster 2, 3, 5 and 6) and shared responses to IFNγ treatment (cluster 4). **c** Boxplots presenting the abundance of various proteins associated with inflammation in control and IFNγ-treated alpha, beta and delta cells. Biological replicates *n* = 3 per group. **d**–**f** Volcano plots illustrating the differential enrichment of proteins between control and IFNγ-treated alpha, beta and delta cells. Significantly regulated proteins (adj. *p*-value < 0.05, |log2 fold change| > 1) are highlighted as black dots. Colored proteins are based on their regulation: alpha- (red), beta- (blue) and delta-specific (green), while black labels are presented in two cell types.

PCA revealed that IFNγ-treated alpha, beta, and delta cell populations could be distinguished from untreated cells along principal component 3 (Fig. 3a and Supplementary Data 1). Analysis of variance (ANOVA) identified significant differences in 67% of all proteins across all conditions (cell type and upon IFNγ treatment) (Supplementary Fig. 4 and Supplementary Data 2). However, the vast majority of them (97%) remained unaltered upon IFNγ treatment alone.

We observed significant overlap of the proteins induced by IFNγ across the three endocrine cell subsets (Fig. 3b), a common responsiveness to this treatment. Key upregulated proteins in all three cell types included Igtp (an IFN-induced gtpase), Stat1 and Stat2 (transcription factors of the IFN pathway), and Tap1 (associated protein to class I MHC processing) (Fig. 3c).

Intriguingly, we also identified cell type-specific protein enrichments following IFNγ treatment (Fig. 3d–f): alpha cells: Gbp8 (another IFN-induced GTPase), Ryr2 (an intracellular Ca^2+^ channel), and Rbms1 (an RNA-binding protein) (Fig. 3d); beta cells: Cxcl10 (a cytokine), Lama2 (a laminin subunit), Fau (a ubiquitin-like protein), and Mfsd8 (an orphan membrane transporter) (Fig. 3e); delta cells: Trim41 and Rnf115 (two E3 ligases related to innate immunity), Elf5 (a transcription factor), and Tuft1 (a regulator of the mTOR pathway) (Fig. 3f).

Our analysis of stress-sensitive proteins (including HSP70/90 family members and PERK pathway factors) revealed consistent expression between control and IFNγ-treated samples within each cell type (Supplementary Fig. 3c). The absence of treatment-induced stress responses—despite baseline variations between cell types—confirms that the observed 4% variance in PC3 specifically reflects IFNγ effects rather than processing artifacts. This validates our experimental approach and strengthens confidence in the biological relevance of IFNγ-mediated changes. Thus, our proteomic profiling successfully captured the fingerprint of IFNγ-induced changes in mouse alpha, beta, and delta cells, providing a novel approach to explore the dynamic proteome in response to inflammatory stimuli.

### Proteome and transcriptome signatures correlate in a cell type-specific manner

While transcriptome and proteome analyses both provide genome-wide characterization of cellular responses to perturbations, mRNAs are known to be imperfect predictors of protein abundance. Typically, Pearson correlation numbers for explained variation in protein concentration by mRNA levels are often around R = 0.4 or R^2^ = 0.16^24,25^, despite high precision between biological replicates in both mRNA and protein measurements^26^.

We performed an omics correlation analysis of our previously acquired RNA-seq data^17^ (in total 17,230 genes) to our proteomic dataset (in total 7285 proteins) with 4481 matching genes across all three cell types (alpha, beta, delta) and two treatment conditions (control, IFNγ-treated) and three biological replicates (*n* = 18). This revealed high intra-replicate mean R-squared values around 0.98 and 0.91 (Supplementary Fig. 5 and Supplementary Data 2), with mean CVs of 9.3% and 21.8% for proteomics and transcriptomics, respectively (Supplementary Fig. 6 and Supplementary Data 2).

**Fig. 4:**
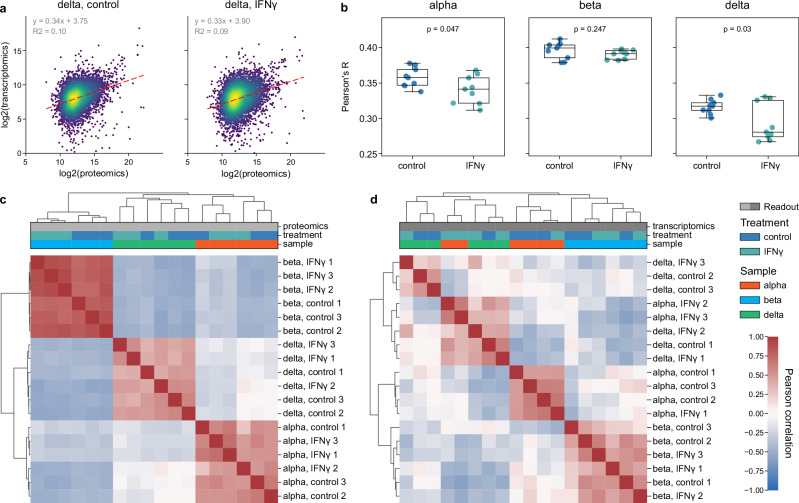
Integration of proteomics and transcriptomics data. **a** Representative cell-specific comparison of triplicate median values between proteomics (*x*-axis) and transcriptomics (*y*-axis) data for delta cells. Linear regressions (red dashed lines) are shown with equations and R-squared values (indicated in respective boxes). **b** All-versus-all replicate comparison between proteomics and transcriptomics for each cell type and treatment conditions. Each dot represents the Pearson correlation value from a unique pairing of one proteomics and one transcriptomics replicate in their respective conditions. Statistical significance was determined using two-sided independent Student’s *t*-tests, with *p*-values < 0.05 considered significant (*). **c**, **d** Proteomics (**c**) and transcriptomics (**d**) sample-sample Pearson correlation coefficient heatmap. Each tile is colored by the R-value of comparing two samples, while rows and columns are sorted by single linkage Euclidean clustering.

To assess the correlation between transcriptomics and proteomics data, we employed linear regression and Pearson correlation analyses. Beta cells showed the highest correlations overall and delta cells the lowest (Fig. 4a and Supplementary Data 1, Supplementary Fig. 7 and Supplementary Data 2). Interestingly, proteome-transcriptome correlations were significantly lower for IFNγ-treated delta and alpha cells compared to controls, but remained unchanged for beta cells (Fig. 4b, Supplementary Fig. 8 and Supplementary Data 2). We hypothesize that this observed difference may result from rapid transcriptional responses to IFNγ signaling, causing a temporary discrepancy between the cell’s proteomic inventory and synthesized mRNAs.

We then investigated whether the cell type and treatment conditions could be distinguished based on cellular proteomics and transcriptomics signatures. Our analysis revealed that proteomics signatures allowed for a clearer separation of cell types (Fig. 4c). Conversely, transcriptomics signatures showed less distinct clustering for cell types (Fig. 4d). These findings highlight that proteomics offers a highly accurate approach regarding cell types, beyond transcriptome sequencing. The observed discrepancies between proteome and transcriptome suggest cell type-specific dynamics in mRNA and protein abundance regulation. An interesting question for the future is to what extent longer IFNγ treatments might lead to closer synchronization of mRNA and protein levels in response to perturbation.

### Determining cell type-resolved phosphoproteome profiles for alpha, beta and delta cells

**Fig. 5:**
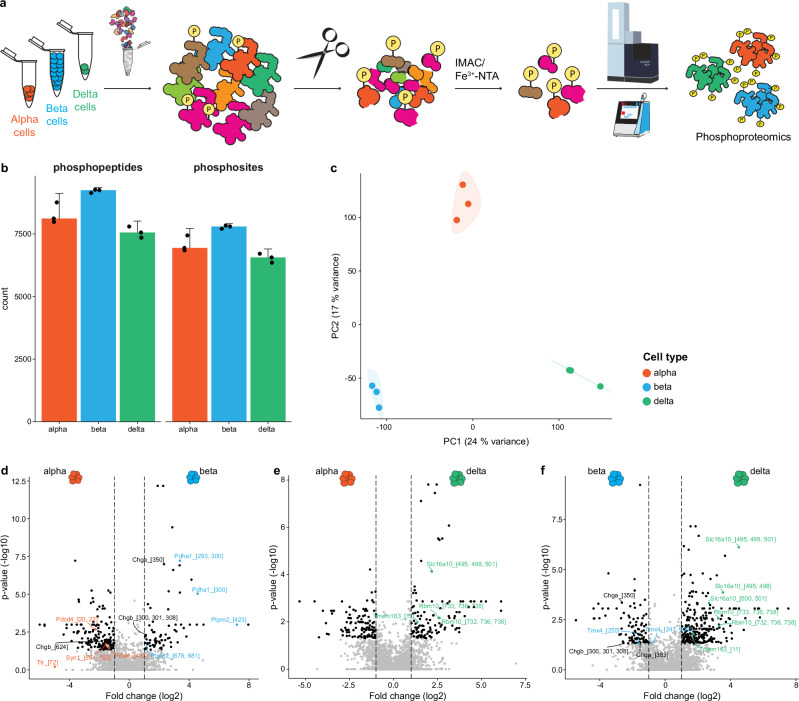
Cell type-resolved phosphoproteomic landscape of mouse alpha, beta and delta cells. **a** Schematic representation outlining the experimental workflow from alpha, beta and delta cells sorted from male mouse islets across the different steps of phosphoproteomic profiling. **b** Quantification of phosphopeptides and phosphosites in alpha, beta and delta cells. Each point corresponds to a pool of islets harvested from 3 different mice. Error bars represent the standard error of the mean (SEM) calculated from *n* = 3 biological replicates. **c** PCA applied to the phosphoproteomic profiles of alpha, beta and delta cells. **d**–**f** Volcano plots showing the differential enrichment of phosphosites in alpha vs beta vs delta cells. Biological replicates *n* = 3 per group. Significantly regulated proteins (adj. *p*-value < 0.05, |log2 fold change| > 1) are highlighted as black dots. Colored proteins are based on their regulation: alpha- (red), beta- (blue) and delta-specific (green), while black labels are presented in two cell types.

Previous phosphoproteome analysis required cell inputs as high as 20 µg protein, corresponding to approximately 100,000 cells, limiting exploration, particularly of alpha and delta cells. Here, we established a new strategy in order to determine the phosphoproteome profile of mouse alpha, beta, and delta cells using low input material (Fig. 5a and Supplementary Data 1). First, we performed our FACS-based approach to sort alpha, beta, and delta cell populations from 12-week-old C57BL/6 mice. Then, we enriched the phosphopeptide fraction using Fe^3+^-NTA to chelate the phosphosites, followed by phosphopeptide profiling. Starting from only 25,000 alpha and beta cells, and 14,000 delta cells, we detected more than 7000 sequence distinct phosphopeptides per endocrine subset (8287 ± 416, 9219 ± 68, and 7570 ± 223 phosphopeptides, for alpha, beta, and delta cells, respectively) (Fig. 5b). Of these, more than 6000 represented unique phosphosites (7077 ± 320, 7780 ± 65, and 6543 ± 179 for alpha, beta, and delta cells, respectively) (Fig. 5b). PCA revealed that the phosphoproteomic profiles of alpha, beta and delta cell populations cluster independently (Fig. 5c).

We conducted comparative analyses to identify phosphosites enriched in alpha, beta or delta cell populations (Fig. 5d–f). Given the lack of data on phosphoproteomics for alpha, beta, and delta cells, we found numerous enriched phosphosites with unexplored functions. Some phosphosites enriched in alpha cells correspond to cell type-specific proteins, like Ttr (Fig. 5d). We also found phosphopeptides enriched in a specific cell type that correspond to proteins also expressed in other endocrine populations, suggesting selective phosphorylation. This is the case for Syn1 (involved in exocytosis), Pdia3/6 (modulators of protein folding) and Pdcd4 (cell death protein), which are expressed in all three cell types but phosphorylated only in alpha cells (Fig. 5d). In beta cells, we observed this for Pdha1 (a glycolytic protein), Ptprn2 (phosphatase found to be a major autoantigen in diabetes), Dstn (a cytoskeleton component), and Tmx4 (a membrane protein) (Fig. 5e). In delta cells, we found enriched phosphosites for Rbm10 (regulator of cell death), Slc16a10 (an amino acid transporter), and Tmem163 (Zn transporter) (Fig. 5f).

Notably, the pan-endocrine markers Chga and Chgb were differentially phosphorylated across cell populations (Fig. 5d–f). These two proteins promote the generation of hormone-containing secretory granules, although their function and regulation are poorly understood^27,28^. Chgb phosphorylated at site 624 is enriched in alpha cells (Fig. 5d, e) when compared to beta cells, where it is mainly phosphorylated at sites 300, 301, and 308 (Fig. 5e, f). In beta cells, Chga is preferentially phosphorylated at sites 350, 383 and 410, 414, 419 (Fig. 5e, f) when compared to delta cells, where it is mainly phosphorylated at site 119 (Fig. 5d, e). These findings suggest that differential phosphorylation of common endocrine proteins, like Chga and Chgb, across cell types could contribute to cell type-specific mechanisms of vesicle biogenesis and release.

## Discussion

In this study, we present a proteomic dataset of murine adult pancreatic alpha, beta and delta cell populations from adult male mice, combining a FACS-based approach with deep proteomics profiling. Our approach not only identified the dynamic proteomic fingerprint of each cell type under basal and inflammatory-like conditions but also resolved their phosphoproteomic profiles for the first time. Mouse pancreatic islets are composed of alpha, beta and delta with beta cells comprising about 75% of the islet population. Previous studies have provided a beta-centric view of the islet proteome, without cell type-specific distinctions^10,11^, limiting our understanding of alpha and delta cell proteomes. Our cell type-specific approach addresses this gap, offering insights into the unique proteomes of these less abundant cell populations^14,17^.

Our previously established sorting strategy enriches pancreatic alpha, beta, and delta-cell fractions to >90% purity, as validated by RNA-seq, single-cell qPCR, and immunohistochemistry (95%, 88% and 95% for alpha, beta, and delta, respectively)^14^. Our RNA-seq data^14^ show negligible *Ghrl* expression in adult α-, β-, and δ-cells, consistent with reports of rare epsilon cells in mature islets^29^. By proteomics, we detected Ghrl only sporadically in delta cells at trace levels, confirming minimal epsilon-cell contamination in our sorted populations (Supplementary Fig. 9 and Supplementary Data 2).

FACS followed by proteomics has been used in the past in other tissues, for instance, to construct an in-depth quantitative proteomic atlas of human skin melanocytes, endothelial and immune cells^30^. In another study, endothelial and immune cells were sorted from the gut of patients with Crohn’s disease^31^. However, these studies used orders of magnitude larger amounts of starting tissues and were not limited in sample input. In contrast, our FACS-based approach, followed by deep proteomics, was readily applicable to mouse pancreatic endocrine cells, which comprise only 2% in the pancreas. Therefore, we were constrained by the scarcity of our biological material, particularly for delta cells. Despite these challenges, we achieved comprehensive proteomic coverage (about 6000 protein groups). This unique dataset therefore provides novel insights into the proteome differences between these specialized endocrine cell types, especially for the understudied delta cells, which are typically difficult to obtain in sufficient quantities for comprehensive proteomic analysis and are therefore understudied. Further, our approach captured the dynamic proteome of alpha, beta and delta cells upon IFNγ exposure, a mediator of autoimmune damage in type 1 diabetes^32^. We also attempted to profile islet cell populations from Non-Obese Diabetic (NOD) mice; however, the inherent fragility of dying beta cells and selection bias toward surviving islets introduced technical limitations. Future studies could employ spatial proteomics on pancreatic sections, though precise endocrine cell isolation would remain challenging. Finally, adapting our approach to human islets would be valuable; however, it falls outside the scope of this study, and we hope our findings inspire further advancements in this area.

Our analysis revealed novel proteins expressed in specific endocrine cell populations. For instance, we detected Gpr158 specifically in beta cells, a finding that aligns with mRNA expression data. This discovery provides new opportunities for studying GPCR function in beta cells, particularly in relation to insulin secretion and cell survival^33^. Due to the lack of properly validated Gpr158 antibodies, this protein has not been analyzed in pancreatic beta cells, and its specific expression opens new avenues to study the role of this new GPCR in beta cells. Whether its recently described ligands osteocalcin^34^ and glycine^22^ in the central nervous system signal on beta cells to modulate insulin secretion or cell survival will be the topic of future studies. Gpr158 can also now be added to the list of beta cell-specific proteins that are also expressed in neurons, such as the transcription factor Nkx6.1^35^, Glutamic Acid decarboxylase (Gad1) and the membrane glycoprotein Bace2^36^. We also identified new pancreatic endocrine cell surface markers, such as Sirpa, a membrane protein that acts on neighboring cells through interactions with CD47^37^. Here, we demonstrate that Sirpa is highly enriched in beta but also delta cells. Sirpa has recently been shown to enhance beta cell viability^23^, and our results suggest potential protective roles in delta cells as well. Finally, our sensitive approach allowed for deep proteome profiling of the less abundant delta cells, revealing enrichment in proteins regulating vesicle exocytosis^38^ (Syt1, Snca) and neuronal-like features (Gap43, Ncam2, Spock1, Spock3, Sema3e), which may explain their unique morphology^39^.

The phosphoproteome dataset we present is the first for pure pancreatic endocrine populations, achieved with limited sample input. We identified cell type-specific phosphorylation of Chga and Chgb, suggesting differential regulation of granule formation across cell types. This adds a new layer to our understanding of islet cell physiology.

Our findings provide novel insights into islet biology by identifying previously uncharacterized proteins differentially expressed in alpha, beta and delta cells, which have been largely underexplored in existing proteomic datasets. As an example, we discovered new cell surface markers such as Sirpa, which can now be utilized to further purify alpha, beta and delta cell populations by FACS. In conclusion, our work provides an in-depth (phospho)proteomic resource of murine adult pancreatic endocrine cells, revealing distinct protein signatures and phosphorylation patterns. This resource lays the foundation for future studies in islet biology, diabetes pathogenesis, and potential therapeutic targets. Moreover, our methodology demonstrates the feasibility of deep proteomic profiling from limited biological samples, with broad implications for biomedical research. Our approach could be extended to translating these findings to human islet cells and advancing single-cell proteomic approaches^40,41^. These efforts will further enhance our understanding of pancreatic endocrine cell biology and its implications for human health and disease.

Our study has some limitations. First, it was conducted exclusively in male mice, and future studies should include female mice to determine whether the findings are generalizable across sexes. Second, alpha, beta and delta cell populations used in the present study are not 100% but about 90% pure. Moreover, we cannot fully exclude some contamination by PP cells. These cells express the hormone PPY; however, this hormone is not cell type-specific^42^ and markers specific for PP cells are not available. However, if some contamination exists, it would be minimal as PP cells are present in pancreatic islets at a very low frequency. Third, the proteomic and transcriptomic datasets were acquired from different mice. However, both datasets were generated using identical standardized experimental protocols and instrumentation. This consistency in methodology helps ensure comparability between the datasets, although biological variation between individuals should be considered when interpreting correlations between protein and transcript levels. Fourth, our in vitro study represents a methodological advance in islet cell proteomics. In vivo proteomic experiments on islets from NOD mice, a model of type 1 diabetes, would increase the pathophysiological strength of our study. Fifth, phosphoproteomics analysis was limited to control conditions and did not include IFNγ-treated cells. This was primarily due to technical constraints specific to the experimental setup. Phosphorylation events occur on a faster timescale (minutes to hours) compared to the transcriptional and proteomic changes we studied (hours to days). Therefore, IFNγ treatment needed to be performed after FACS sorting to capture these rapid phosphorylation dynamics accurately. However, after FACS sorting, the limited cell numbers obtained for alpha and delta cells made them unstable under IFNγ treatment conditions, preventing reliable phosphoproteomic analysis. Finally, while our identification of ~7000 phosphosites per cell type advances endocrine phosphoproteomics, three barriers currently limit functional insights: technical sensitivity thresholds; unavailable phospho-antibodies; and computational challenges with disordered regions in structure predictions.

## Methods

### Animals

Twelve-week-old C57BL/6JRj male mice were purchased from Janvier Labs (Saint Berthevin, France). Mice were kept on a 12:12 light-dark cycle and provided with water and food ad libitum. They were euthanized by cervical dislocation. The animal studies complied with the ARRIVE guidelines and were conducted in accordance with the EU Directive 2010/63/EU for animal experiments and with regard to specific INSERM guidelines. All the experiments were approved by the Ethical Committee of Paris Cité University and the French Ministry of Higher Education and Research (#16376-2017122210502504).

### Isolation, culture and treatment of mouse pancreatic islets

Mouse islet isolation was performed as described^17^. Briefly, type V collagenase (#C9263, Sigma-Aldrich, St. Louis, MO, USA) was injected into the common bile duct while the ampulla of Vater was clamped. The inflated pancreas was collected and digested for 20 min at 37 °C. Digested pancreas was resuspended in HBSS (#14025, ThermoFisher Scientific, Waltham, MA, USA), and islets were then handpicked. They were cultured for 17 h in RPMI 1640 (#61870-010, ThermoFisher Scientific) with 10% fetal calf serum (FCS CVFSVF00-01, Eurobio, Les Ulis, France) and penicillin/streptomycin (#15140122, ThermoFisher Scientific). Next, islets were treated with or without IFNγ (5 ng/ml) for 20 h (#485-MI-100, R&D Systems, Minneapolis, MN, USA). For IFNγ treatment, pooled islets from 3 mice per condition were cultured for 20 h with or without 50 ng/ml IFNγ prior to FACS sorting.

### Flow cytometry and sorting

Islets were dispersed in single-cell suspensions using the Neural Tissue Dissociation Kit (#130-092-628, Miltenyi Biotec, Bergisch Gladbach, Germany). Cell surface staining was performed as described^17^. Briefly, dispersed islet cells were centrifuged and resuspended in FACS medium (HBSS 10% Fetal Calf Serum) with antibodies for 15 min at 4 °C in the dark. The following antibodies were used: CD31 (1:100, #102522, Biolegend), CD45 (1:100, #103132, Biolegend), CD235a (1:100, #349105, Biolegend), CD24 (1:100, #101840, Biolegend), CD71 (1:100, #113806, Biolegend), CD49f (1:200, #313612, Biolegend), CD81 (1:100, #740060, BD Biosciences), SIRPA (1:100, #144008, Biolegend), EPCAM (1:100, #118210, Biolegend). Then, cells were rinsed and resuspended in FACS medium (HBSS 10% Fetal Calf Serum) with propidium iodide (1/4000, #P4864, Sigma-Aldrich). For each antibody, the optimal dilution was determined by titration. Cell sorting was carried out using a FACS Aria III (BD Biosciences), and data were analyzed using FlowJo™ Software 10.6.1 (RRID:SCR_008520, BD Biosciences). FACS-sorted cells were washed twice with cold PBS, centrifuged at 500×*g* and flash-frozen until further processing.

### RNA extraction, reverse transcription, and RT-qPCR

RNA extraction was performed using the RNeasy Micro kit (#74004, Qiagen) according to the instructions of the manufacturer on cells sorted in RLT buffer (#74004, Qiagen, Hilden, Germany). cDNAs were obtained using the Maxima First Strand cDNA synthesis Kit (#K1642, ThermoFisher Scientific) according to the protocol of the manufacturer. RT-qPCR was performed with Power SYBR Green Master Mix (#4367659 ThermoFisher Scientific) on a QuantStudio 3 machine (ThermoFisher Scientific). Primers sequences (Eurofins France, Nantes, France) are presented in Table 1. The relative gene expression was assessed according to the 2^−∆Ct^ × 100 (expressed as % of Ppia gene expression).

**Table 1:** Sequences of the primers employed for qPCR

Gene	Sequence forward	Sequence reverse
*Ins1*	CAGAGACCATCAGCAAGCAG	GGGACCACAAAGATGCTGTT
Gcg	TGAAGACAAACGCCACTCAC	TGACGTTTGGCAATGTTGTT
Sst	TCCGTCAGTTTCTGCAGAAGTCTC	GTACTTGGCCAGTTCCTGTTTCCC
*Amy1*	GTTATCCGCAAGTGGAATGG	TCGCTGATTGTCATGGTTGT
*Spp1*	ATCTCACCATTCGCA	TGTAGGGAGGATTGGAGTGAAA
*Cpa1*	GGAGGAGTTGGAGCATTGA	AGAATGCTTTCACGGACTGG
*Ppia*	CAGGTCCTGGCATCTTGTCC	TGCTTGCTGGTCTTGCCATTCC

### Sample preparation for proteomic profiling

Samples were prepared as described^43^. Briefly, cell pellets were resuspended and lysed in 2% sodium deoxycholate (SDC), 100 mM Tris, pH 8.5. For reduction and alkylation of disulfide bonds, 10 mM TCEP (tris(2-carboxyethyl)phosphine) and 40 mM chloroacetamide were added to the buffer. Samples were heated for 10 min at 95 °C while shaking. Protein lysate was sonicated for 10 min at maximum energy in 30 s on and off (Bioruptor, Diagenode, Seraing, Belgium). Trypsin (Sigma-Aldrich) and LysC (FUJIFILM Wako) were added for protein digestion in a 1:25 enzyme:protein ratio. Proteins were digested at 37 °C overnight while shaking (at 800 rpm). Peptides were acidified by 1% trifluoroacetic acid (TFA) in isopropanol and purified by SDB-RPS (styrene divinylbenzene-reversed phase sulfonate) cleanup. Peptides were washed twice with 1% TFA/isopropanol and twice with 1% TFA/ddH_2_O before elution with 5% NH_3_ in 80% acetonitrile (ACN). Dried eluates were reconstituted in water with 2% ACN and 0.1% TFA.

### Sample preparation for phosphoproteomic profiling

For phosphoproteomics, islet cells were prepared and sorted as described above. Cells were next, washed twice with cold PBS, centrifuged at 500×*g* and flash-frozen until further processing. Cell pellets were resuspended and lysed in 60 mM TEAB (Triethylammoniumbicarbonat), pH 8.5, with 10% ACN and 0.013% n-Dodecyl-Beta-Maltoside (DDM). Disulfide bonds were reduced and alkylated by the addition of 5 mM TCEP and 25 mM CAA. Samples were lysed at 76 °C for 20 min while shaking at 1200 rpm, and sonicated at maximum energy for 8 cycles at 15 s on and 30 s off (Bioruptor, Diagenode, Seraing, Belgium). Trypsin (Sigma-Aldrich) and LysC (FUJIFILM Wako) were added for protein digestion in a 1:25 enyzme:protein ratio. Proteins were digested at 37 °C overnight. Digestion was stopped by 0.5% TFA and subsequent centrifugation at max speed for 5 min to remove cell debris. About 50 ng were used for proteomic measurements for normalization on the proteome level. The remaining peptide material was enriched for phosphopeptides with the AssayMAP bravo robot (Agilent)^44^ using 5 μl Fe(III)–nitrilotriacetic acid AssayMap cartridges as described. However, elution was performed using 500 mM NH4H2PO4, pH 4.5, 0.01% DDM. Phosphopeptides were directly eluted by a customized software script into prepared Evotips (StageTips, Evosep) containing 150 µl buffer A (99.9% H_2_O, 0.1% FA) and followed by centrifugation at 700×*g* for 2 min.

### Peptide loading onto C-18 tips

C-18 tips (Evotip Pure, Evosep) were loaded with the Bravo robot (Agilent), by activation with 1-propanol, washing twice with 50 μl buffer B (99.9% ACN, 0.1% FA), activation with 1-propanol and two wash steps with 50 μl buffer A (99.9% H_2_O, 0.1% FA). In between, Evotips were spun at 700 × g for 1 min. For sample loading, Evotips were prepared with 70 μl or 170 μl (phosphoproteomics) buffer A and a short spin at 700×*g*. After sample loading, Evotips were washed with 50 μl buffer A and stored with 150 μl buffer A after a short spin at 700×*g* at 4 °C until MS acquisition.

### Mass spectrometric acquisition by LC-MS

The reconstituted samples were measured with an EASY-nLC 1200 (ThermoFisher Scientific) nano-flow liquid chromatography system (nLC). A home packed column (50 cm, 75 µm i.d.) with a pulled emitter tip, packed with 1.9 µm C18-coated porous silica beads (Dr. Maisch, Ammerbuch-Entringen, Germany) was used at 60 °C and with a constant flow of 300 nl/min. Mobile phases A and B were water with 0.1% formic acid (v/v) and 80/20/0.1% ACN/water/formic acid(v/v/vol), respectively. In 120-min gradient, peptides were separated with a linear gradient from 3 to 30% buffer B within 95 min, followed by an increase to 60% buffer B within 5 min, washed at 95% within 10 min and re-equilibrated for 10 min. The nLC was coupled to a trapped-ion mobility spectrometry quadrupole time of flight mass spectrometer (timsTOF Pro 2, Bruker Daltonics, Bremen, Germany) with a nano-electrospray ion source (CaptiveSpray, Bruker Daltonics). The mass spectrometer was operated in dia-PASEF mode using the 16 dia-PASEF scan acquisition scheme (standard scheme)^45^. The method covered a m/z range from 400 to 1200 and ion mobility of 0.6 to 1.6 Vs cm^−^^2^. All other settings were standard as described^40^. Briefly, ion accumulation and ramp time in the dual TIMS analyzer were set to 100 ms each, and the collision energy was increased linearly as a function of mobility starting from 20 eV at 1/K0 = 0.6 Vs cm^−2^ to 59 eV at 1/K0 = 1.6 Vs cm^−2^.

For phosphoproteomic measurements, an Evosep One system was coupled to a timsTOF Ultra (Bruker Daltonics). The Whisper20 samples per day method was used with the Aurora Elite CSI third generation 15 cm and 75 µm ID (AUR3-15075C18-CS IonOpticks, Australia) at 50 °C inside a nano-electrospray ion source (Bruker Daltonics) at 1400 V. The mobile phases were 0.1% formic acid in LC–MS-grade water (buffer A) and 99.9% ACN/0.1% FA (buffer B). The timsTOF SCP was operated with an optimal dia-PASEF method generated with our Python tool py_diAID^46^. The method consisted of twelve dia-PASEF scans and two TIMS ramps, while covering a m/z range from 400 to 1450 and an ion mobility of 0.75 to 1.45 Vs cm^−^^2^. The mass spectrometer was operated in high sensitivity mode, and the collision energy was increased linearly as a function of mobility, starting from 20 eV at 0.6 Vs cm^−^^2^ to 25 eV at 0.85 Vs cm^−^^2^ to 54 eV at 1.17 Vs cm^−^^2^ and at the end to 60 eV at 1.5 Vs cm^−^^2^. For proteome normalization, proteomic samples were acquired in dia-PASEF using twelve dia-PASEF scans and two TIMS ramps covering a m/z range from 350 to 1200 and ion mobility of 0.7 to 1.3 Vs cm^−^^2^. The collision energy was increased linearly as a function of mobility starting from 20 eV at 1/K0 = 0.6 Vs cm^−2^ to 59 eV at 1/K0 = 1.6 Vs cm^−2^. All other settings were used as described above.

### Raw data analysis with DIA-NN for proteomic analysis

Mass spectrometry raw data for proteomic analysis were searched using the DIA-NN software^47,48^ (version 1.8.1) using the library-free search and the mouse Uniprot databases (UP000000589_10090 with isoforms, June 24, 2015). In short, a deep-learning module, match-between-runs (MBR) and heuristic protein inference (‘--relaxed-prot-inf’) were enabled. N-terminal methionine excision and carbamidomethylation were set as fixed modifications, ‘IDs, RT & IM profiling’ was used for library generation, ‘robust LC (high accuracy)’ for quantification and ‘Global’ for cross-run normalization. One miss cleavage was allowed. The ‘pg_matrix.tsv’ output file was used for further data analysis.

### Raw data analysis with Spectronaut for phosphoproteomics

Mass spectrometry raw files for phosphoproteomics were processed with Spectronaut (version 17.4.) using direct DIA analysis (dDIA) with standard settings. In brief, the MS/MS spectra were matched to in-silico derived fragment mass values of tryptic peptides from a reference mouse proteome (Uniprot, UP000000589_10090 with isoforms, June 24, 2015). A maximum of two missing cleavages was allowed, and the required peptide sequence length was 7 to 35 amino acids. Carbamidomethylation of cysteine residues was set as a fixed modification, and methionine oxidation and acetylation of protein N termini as variable modifications with a maximum of 5 per peptide. The false discovery rate (FDR) was controlled at less than 1% for peptide spectrum matches and protein group identifications.

For phosphoproteomic analysis, the raw data were processed with Spectronaut (version 17.4.) using dDIA as described above. Phospho (STY) was set as additional variable modification Precursor filter was set to 3 to 25 best fragments per peptide. Normalization filter type was set to modification type filter of selected Phospho (STY) and enabled PTM localization function with a probability cutoff of 0. Spectronaut output tables were collapsed to phosphosite tables by the peptide collapse (version 1.4.1) plug-in tool for Perseus^49^, using default settings and a localization cutoff of 0.75 (class I sites)^50^.

### Proteome normalization of phosphoproteomics data

To obtain regulated phosphosites that are corrected for proteome regulation, multiple analysis steps were performed. First, each precursor was assigned to a phosphosite id using the phosphosite probabilities of the Spectronaut output tables. Differential expression analysis of the phosphosites was performed by the MS-EmpiRe package^51^, with ‘id.col’ as precursor and ‘prot.id.col’ as phosphosite_id. The same MS-EmpiRe analysis was done with the unenriched proteome dataset as well. Subsequently, the differential expression results of the phospho and the unenriched proteome dataset were mapped by subtracting the log2 fold changes (FCs) of the proteome data from the phospho data (e.g., if a phosphosite has a FC of 2, but the corresponding protein also has a FC of 2, the corrected FC will be 0, meaning that phosphorylation stoichiometry did not change between conditions). Further, a heuristic dampening of the *p*-value significance of the phospho dataset was performed by exponentially increasing the *p*-value (thereby making it less significant) as a function of the FC differences via the equation ${10}^{{\log }_{10}\left({p}_{{ptm}}\right)\cdot {r}_{{FC}}}$, with ${p}_{{ptm}}$ as the *p*-value to be dampened and ${r}_{{FC}}$ as the dampened log2 FC divided by the original log2 FC. If protein regulation strength is the same or stronger than the phosphosite regulation, this results in a *p*-value of 1. While this approach is heuristic, it provides a conservative estimate for phosphosite regulation, as the dampening function was exponential on the logged *p*-values, giving order or magnitude already decreases for small shifts.

### Proteomics downstream statistical data analysis

Bioinformatic analysis and visualization were performed in either Python (Jupyter Notebook), Perseus^49^ (v1.6.7.0) or the R statistical computing environment (version 2023.09.1).

### Omics data analysis

18 samples consisting of ‘control’ or ‘IFNγ’ treated alpha, beta or delta cells, in biological triplicates, were used for the analysis. Proteomics and transcriptomics datasets were aggregated using custom Python code. Proteomics, transcriptomics and phosphoproteomics data were obtained as raw reads and log2 scaled prior to all other calculations. Numerical operations were performed using the Numpy package^52^ (version 1.26.2). ENSEMBL-IDs from transcriptomics data were mapped to canonical UniProt IDs (biomart 0.9.2), which were then matched to UniProt IDs from transcriptomics data. Only overlapping genes or protein groups were considered for the analysis (4481 genes). Correlation between proteomics and transcriptomics data was assessed for each possible replicate combination between the two analysis methods. To assess inter-replicate correlation via simple linear regression (sklearn^53^, version 1.3.0), Pearson correlation (R) and coefficients of determination (R-squared) values were calculated for each comparison. The results were compared between cell types using two-sided independent *t*-tests (scipy^54^, version 1.11.4) (Fig. 4, Supplementary Fig. 8). To assess sample-sample correlation via clustering and heatmaps, proteomics and transcriptomics datasets were processed as follows: (1) Gaussian imputation to eliminate missing values: missing values for each feature are imputed by randomly drawing from a normal distribution N(μ, σ), where σ is the respective feature’s standard deviation s*0.3, and μ is the feature’s mean x̄ - 3*s, (2) features were scaled and centered by subtracting x̄ from each value and dividing by s (z-scoring), (3) Pearson correlations between each sample were computed using Pandas^55^ (version 2.1.4). Clustering and dendrograms were computed via scipy’s ‘linkage’ function with standard settings (linkage = ‘single’, metric = ‘euclidean’). Seaborn^56^ (version 0.14.0.dev0) was used to visualize correlation heatmaps and annotate samples.

### Reporting summary

Further information on research design is available in the Nature Portfolio Reporting Summary linked to this article.

## Supplementary information

Supplementary Information

Description of Additional Supplementary Files

Supplementary Data 1

Supplementary Data 2

Reporting Summary

## Data Availability

The numerical source data for the graphs in the figures and Supplementary figures can be found in Supplementary Data 1 and Supplementary Data 2 files, respectively. All mass spectrometry raw data, libraries, and outputs from each particular search engine analyzed in this study have been deposited to the ProteomeXchange Consortium via the PRIDEpartner repository. Project accession: PXD055289. Datasets for this analysis are available as Supplemental Data.

## Code availability

Code is deposited on GitHub (https://github.com/MannLabs/IsletsOmics.git).
