# Supplementary material for: Decoding adult murine pancreatic islet cell diversity through cell type-resolved proteomics and phosphoproteomics
Source: Commun Biol. 2025 Oct 17;8:1483. doi: 10.1038/s42003-025-08918-8 (PMC12534492; doi:10.1038/s42003-025-08918-8)
Supplement: Supplementary file 1 — Supplementary Information [file 42003_2025_8918_MOESM1_ESM.pdf]

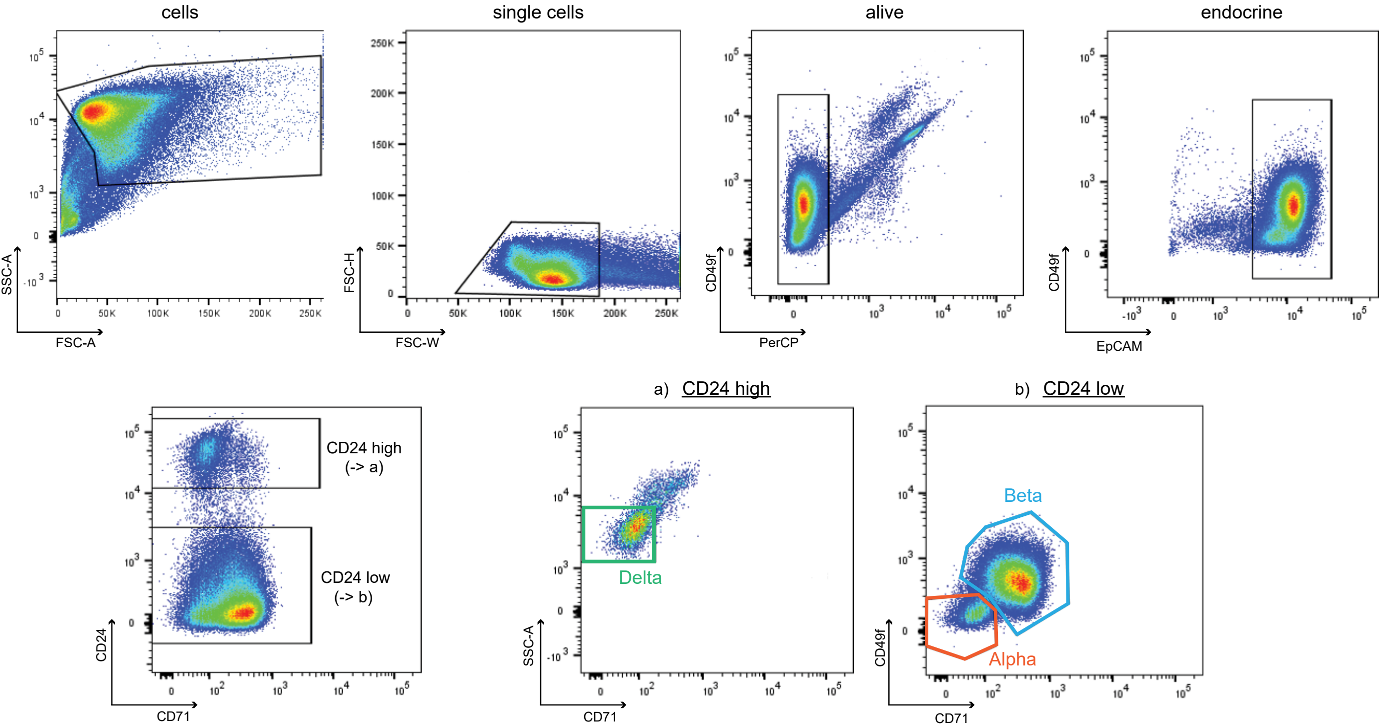

Supplementary Figure 1: Representative flow cytometry plots of dispersed islet cells from adult C57BL/6 mice stained with EpCAM, CD49f, CD24, and CD71.

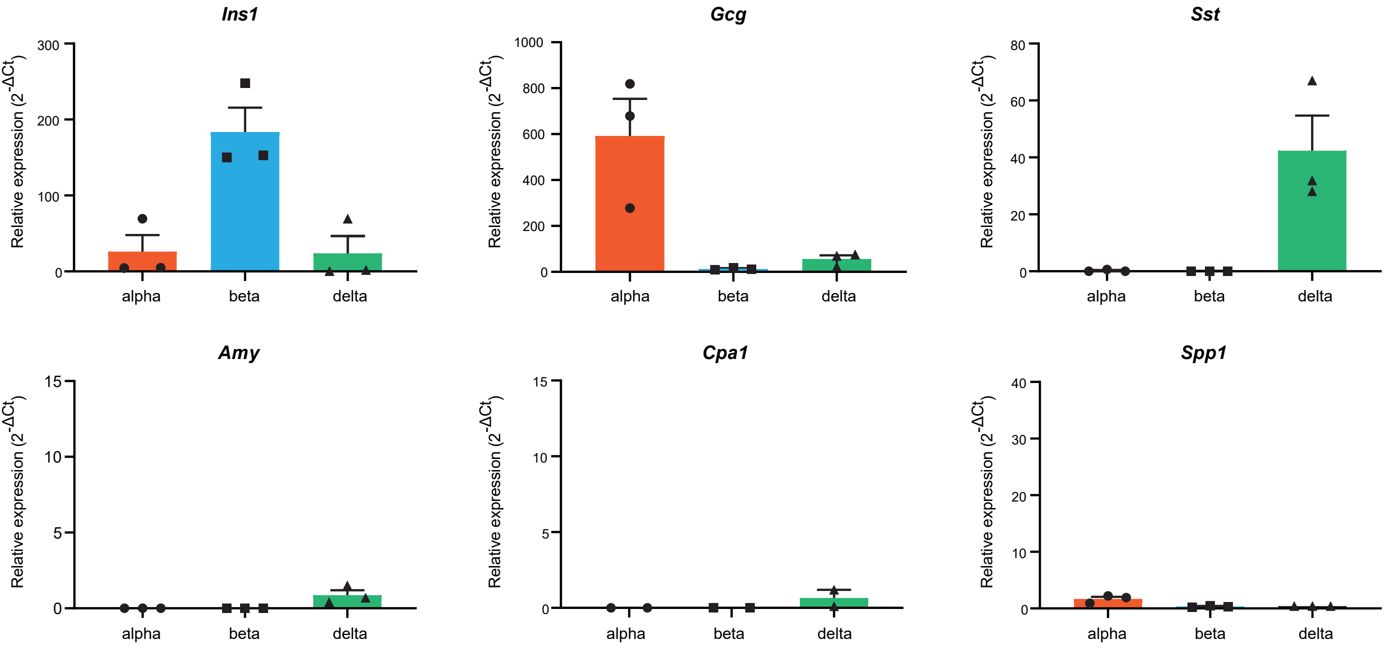

Supplementary Figure 2: Quality control of sorted alpha, beta and delta cell populations analysed by RT-qPCR for alpha (Gcg), beta (Ins1), delta (Sst), acinar (Amy and Cpa1) and ductal (Spp1) cells.

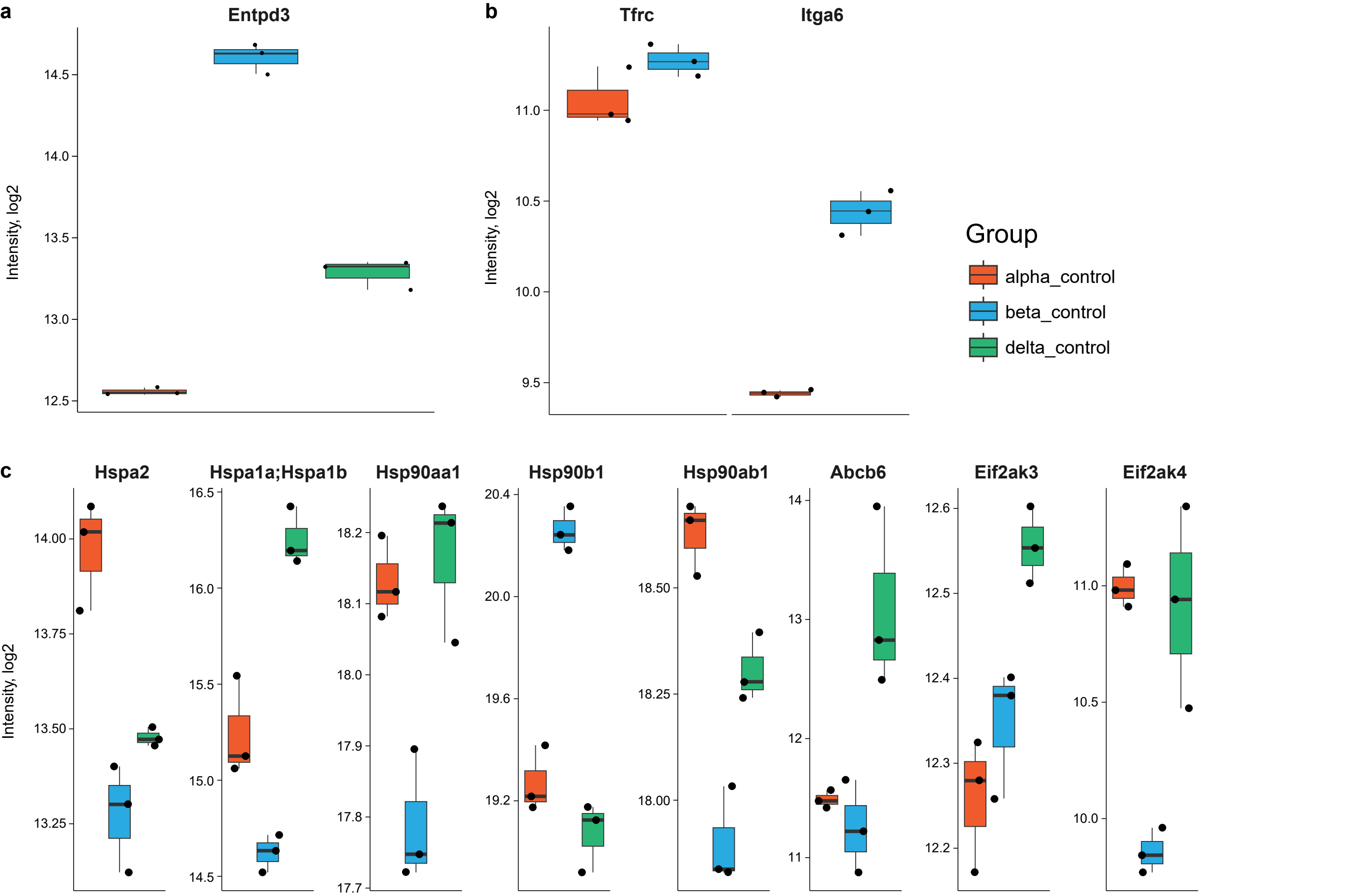

Supplementary Figure 3: Protein expression of (a) Entpd3, (b) surface enrichment markers Tfrc (CD71) and Itga6 (CD49), and (c) key stress makers across the three different cell types.

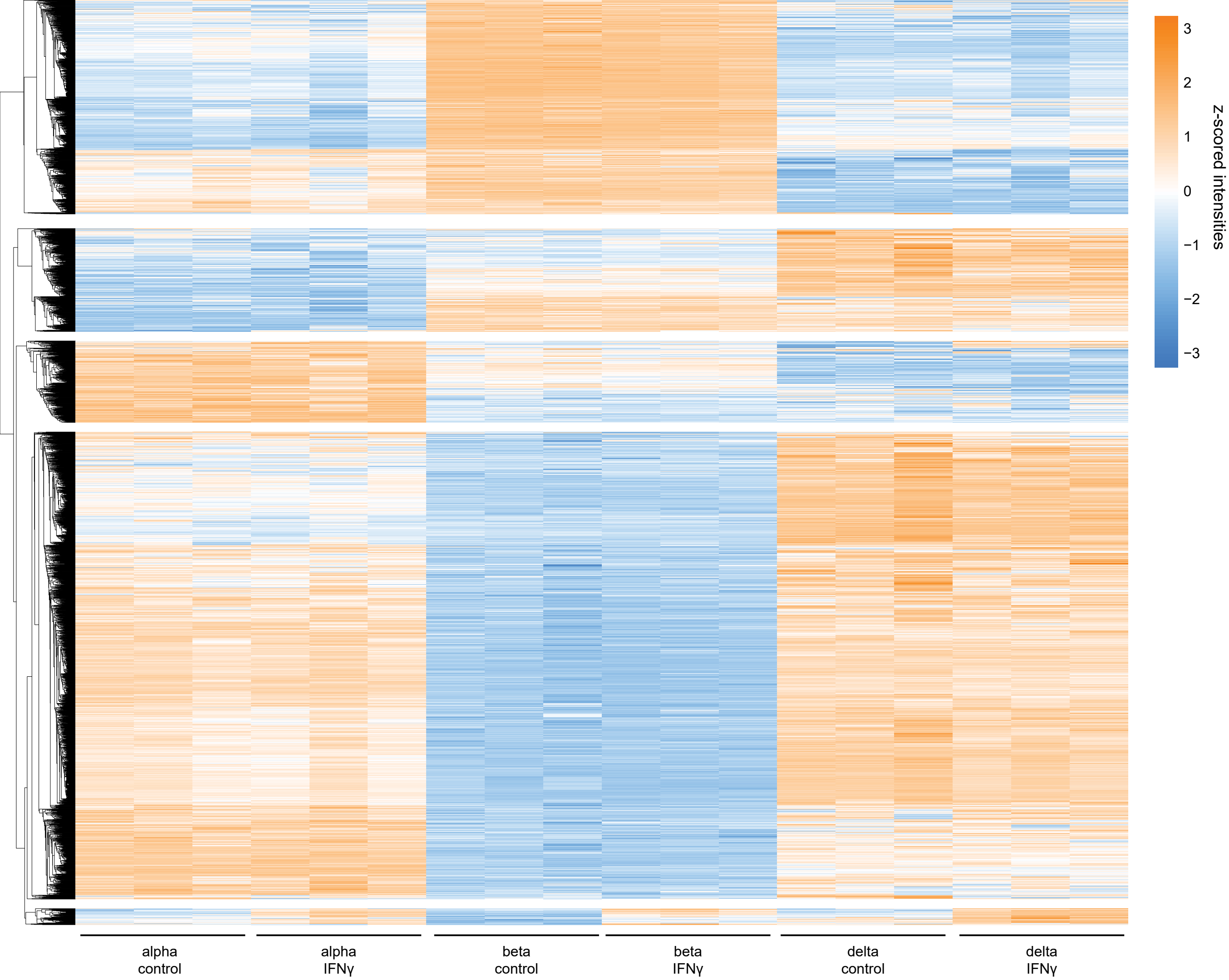

Supplementary Figure 4: Heatmap of protein expression as z-score per protein across all samples. ANOVA significant ($p<0.01$) proteins are displayed in five categories according to hierarchical clustering. The majority of clusters is cell type-driven, while only one cluster is treatment dependent.

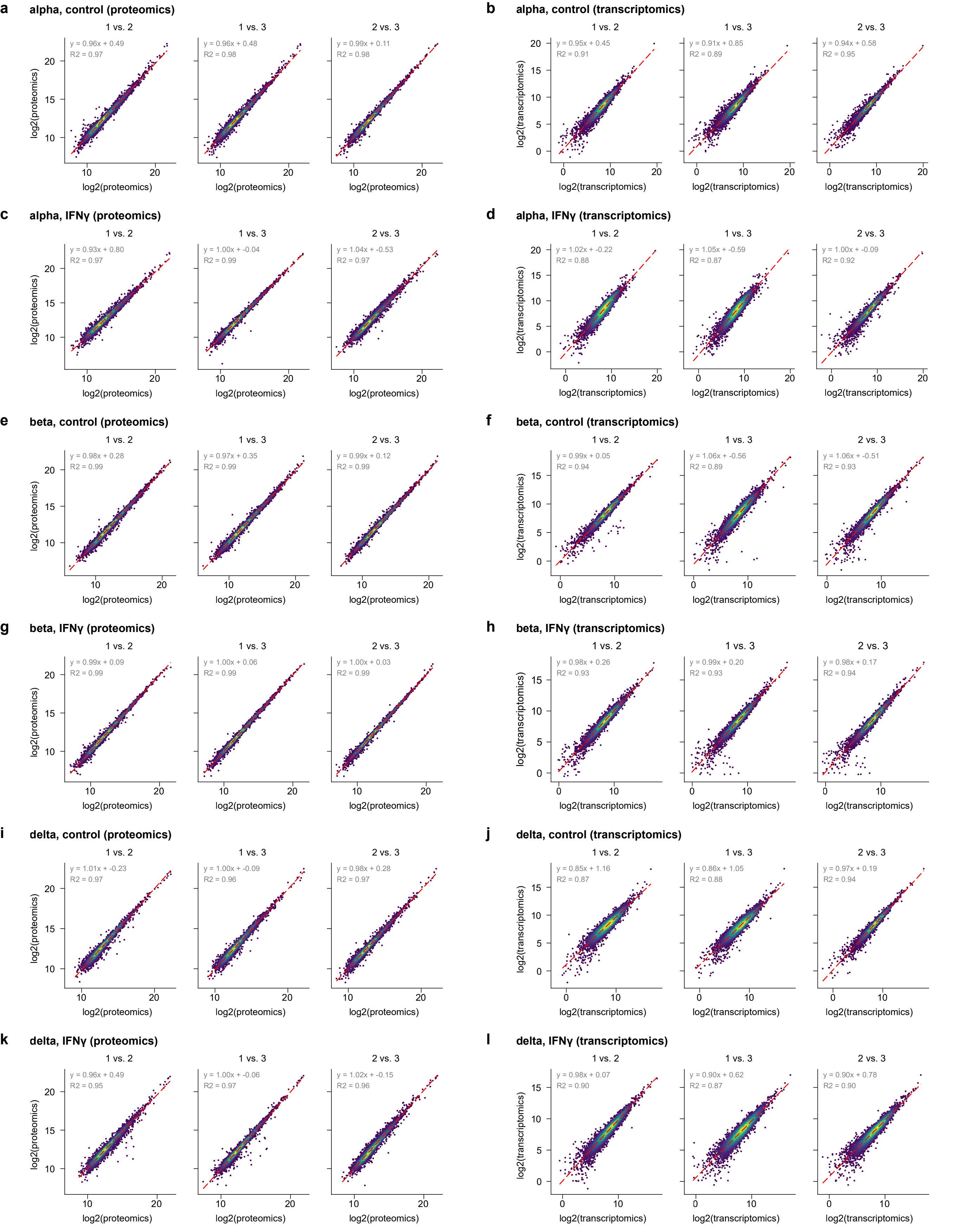

Supplementary Figure 5: Intra-replicate correlations (log2-transformed) of proteomics (a, c, e, g, i, k) and transcriptomics (b, d, f, h, j, l) measurements (n=3 each). Facet titles '1 vs. 2', '1 vs. 3', '2 vs. 3' indicate the respective replicate comparison. Simple linear regression (red, dashed line) was fitted with the equation shown in the respective tile.

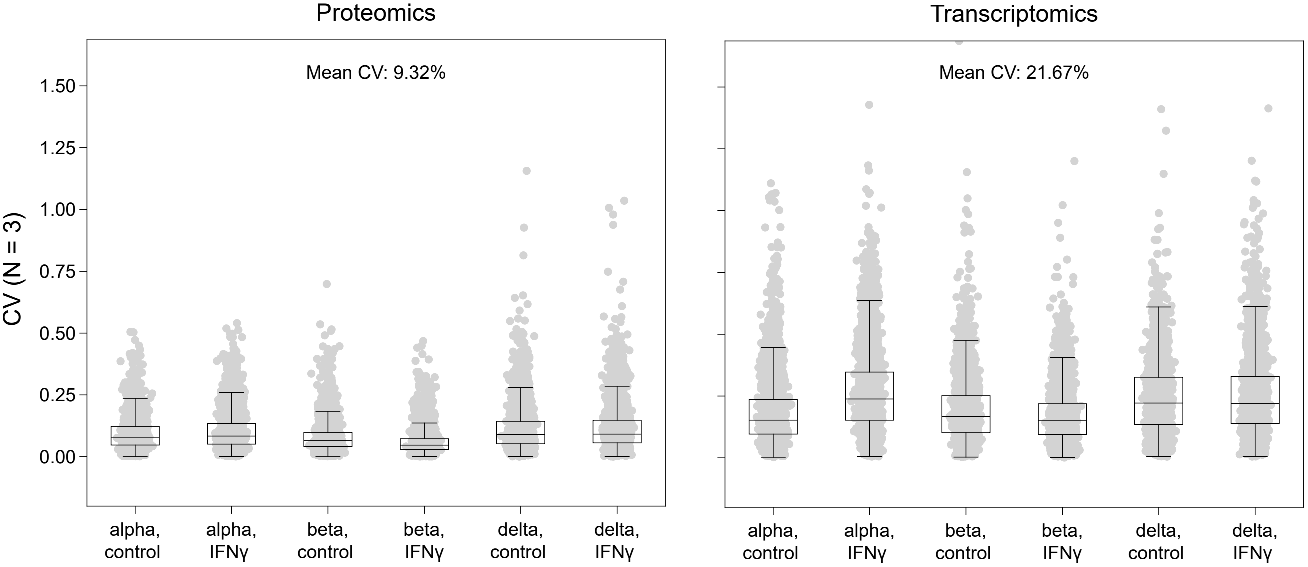

Supplementary Figure 6: Intra-replicate coefficients of variation (CV) for proteomics and transcriptomics data, respectively (n=3 each). 3,273 overlapping genes of proteomics and transcriptomics with no missing values in any replicate were considered for each condition (cell type and treatment). Mean CV was 9.31% for proteomics (left panel) and 21.78% for transcriptomics (right panel).

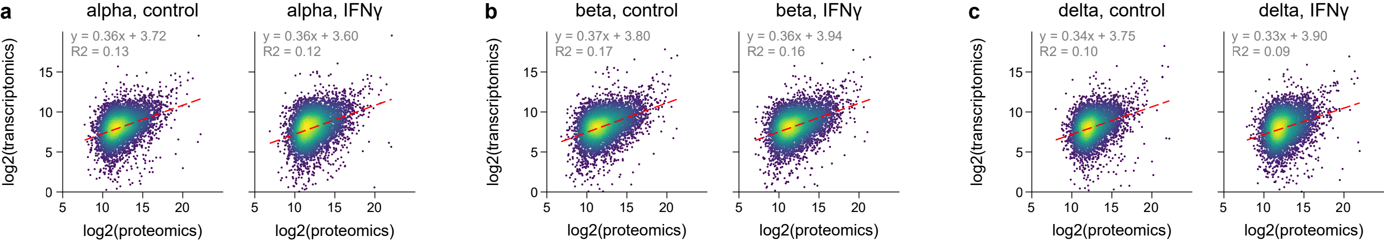

Supplementary Figure 7: Alpha (a), beta (b) and delta (c) cell specific comparison of triplicate median values between proteomics (x-axis) and transcriptomics (y-axis) data. Linear regressions (red dashed lines) are shown with equations and R-squared values (indicated in respective boxes). Same as Fig. 5a for all cell types.

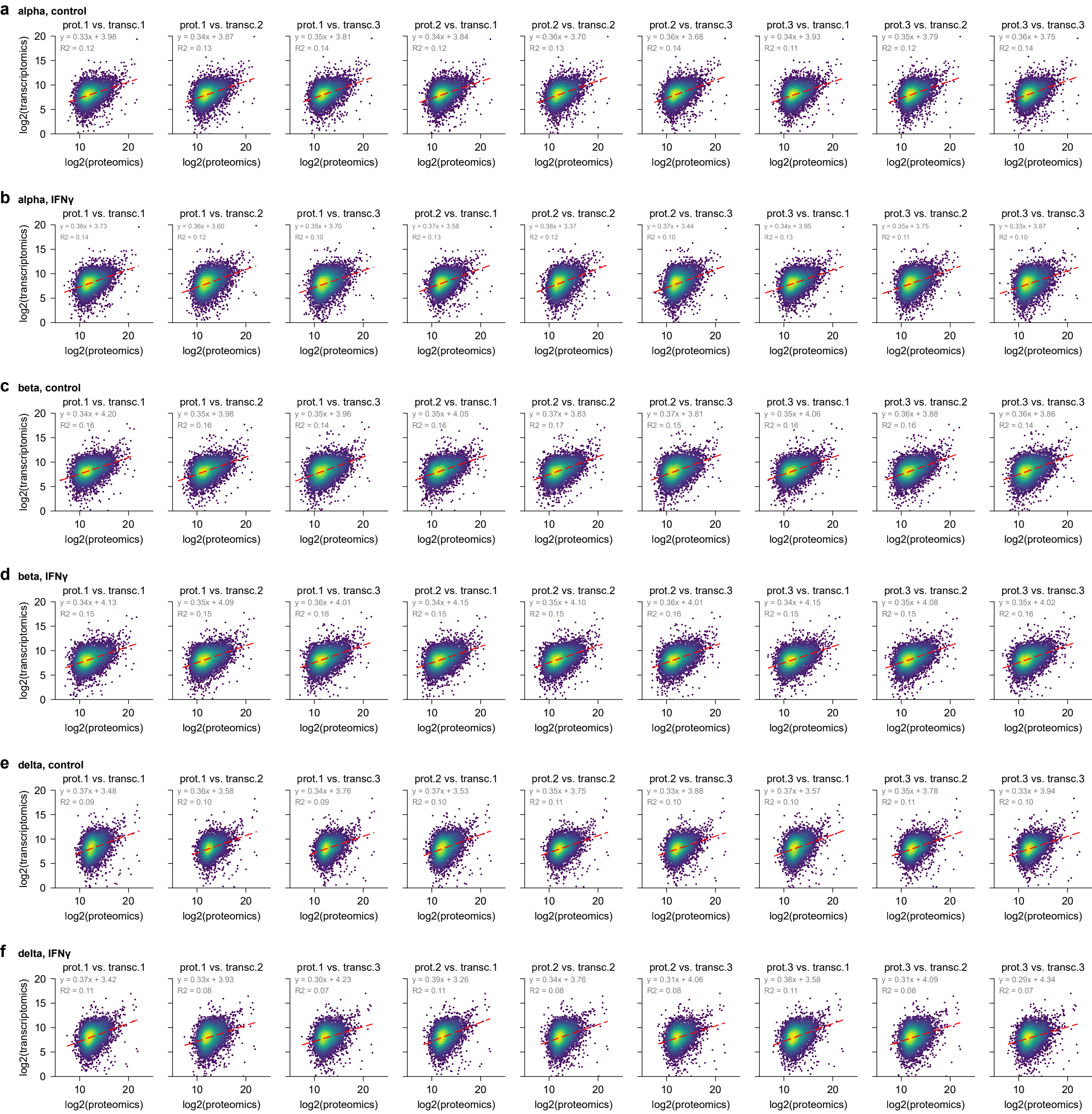

Supplementary Figure 8: Proteomics versus transcriptomics inter-replicate comparison. Each possible combination between omics techniques was evaluated, resulting in nine unique comparisons (column 1vs.1, 1vs.2, etc.) for each cell-type-treatment condition (a - f). Log2-transformed values are shown. Simple linear regression (red, dashed lines) was fitted with the equation shown in the respective tile. Note that these R2 values correspond to the squared Pearson's R values from Fig. 4b.

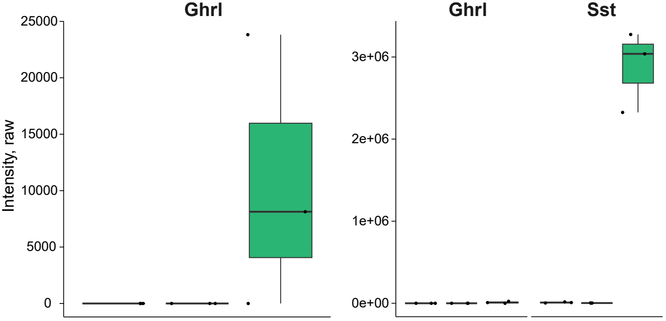

Supplementary Figure 9: Protein expression Ghrl against Sst across the three different cell types to evaluate purity of cell sorting.
