# Supplementary material for: Decoding adult murine pancreatic islet cell diversity through cell type-resolved proteomics and phosphoproteomics
Source: Commun Biol. 2025 Oct 17;8:1483. doi: 10.1038/s42003-025-08918-8 (PMC12534492; doi:10.1038/s42003-025-08918-8)
Supplement: Supplementary file 2 — Description of Additional Supplementary Files [file 42003_2025_8918_MOESM2_ESM.pdf]

Description of Additional Supplementary Files

File name: Supplementary Data 1

Description: Source data for main figures

File name: Supplementary Data 2

Description: Source data for supplementary figures
