# Supplementary material for: Decoding adult murine pancreatic islet cell diversity through cell type-resolved proteomics and phosphoproteomics
Source: Commun Biol. 2025 Oct 17;8:1483. doi: 10.1038/s42003-025-08918-8 (PMC12534492; doi:10.1038/s42003-025-08918-8)
Supplement: Supplementary file 5 — Reporting Summary [file 42003_2025_8918_MOESM5_ESM.pdf]

nature portfolio

Corresponding author(s): Raphael Scharfmmann and Matthias Mann

Last updated by author(s): Dec 9, 2024

Reporting Summary

Nature Portfolio wishes to improve the reproducibility of the work that we publish. This form provides structure for consistency and transparency in reporting. For further information on Nature Portfolio policies, see our Editorial Policies and the Editorial Policy Checklist.

Statistics

For all statistical analyses, confirm that the following items are present in the figure legend, table legend, main text, or Methods section.

n/a	Confirmed	
☐	☒	The exact sample size (*n*) for each experimental group/condition, given as a discrete number and unit of measurement
☐	☒	A statement on whether measurements were taken from distinct samples or whether the same sample was measured repeatedly
☐	☒	The statistical test(s) used AND whether they are one- or two-sided<br>*Only common tests should be described solely by name; describe more complex techniques in the Methods section.*
☐	☒	A description of all covariates tested
☐	☒	A description of any assumptions or corrections, such as tests of normality and adjustment for multiple comparisons
☐	☒	A full description of the statistical parameters including central tendency (e.g. means) or other basic estimates (e.g. regression coefficient) AND variation (e.g. standard deviation) or associated estimates of uncertainty (e.g. confidence intervals)
☐	☒	For null hypothesis testing, the test statistic (e.g. *F*, *t*, *r*) with confidence intervals, effect sizes, degrees of freedom and *P* value noted<br>*Give P values as exact values whenever suitable.*
☐	☒	For Bayesian analysis, information on the choice of priors and Markov chain Monte Carlo settings
☐	☒	For hierarchical and complex designs, identification of the appropriate level for tests and full reporting of outcomes
☐	☒	Estimates of effect sizes (e.g. Cohen's *d*, Pearson's *r*), indicating how they were calculated

Our web collection on statistics for biologists contains articles on many of the points above.

Software and code

Policy information about availability of computer code

Data collection	Mass spectrometry data were acquired with Bruker HyStar, timsControl and Evosep One Software.
Data analysis	Raw data analysis: DIA-NN software (version 1.8.1) and Spectronaut (version 17.4.). Data was analyzed by custom code in R and Python which will be available and shared upon request.

For manuscripts utilizing custom algorithms or software that are central to the research but not yet described in published literature, software must be made available to editors and reviewers. We strongly encourage code deposition in a community repository (e.g. GitHub). See the Nature Portfolio guidelines for submitting code & software for further information.

Data

Policy information about availability of data

All manuscripts must include a data availability statement. This statement should provide the following information, where applicable:

- Accession codes, unique identifiers, or web links for publicly available datasets
- A description of any restrictions on data availability
- For clinical datasets or third party data, please ensure that the statement adheres to our policy

All mass spectrometry raw data, libraries, and outputs from each particular search engine analyzed in this study have been deposited to the ProteomeXchange Consortium via the PRIDEpartner repository. Project accession: PXD055289, username: reviewer_pxd055289@ebi.ac.uk, password: f51EDgz88h5X (https://www.ebi.ac.uk/pride/archive/projects/PXD055289/private).

Datasets for this analysis are available at [https://datashare.biochem.mpg.de/s/SNsrzb6WJF1Knaq], all code is deposited on GitHub: [https://github.com/vbrennsteiner/islets_code.git].

Research involving human participants, their data, or biological material

Policy information about studies with human participants or human data. See also policy information about sex, gender (identity/presentation), and sexual orientation and race, ethnicity and racism.

Reporting on sex and gender	N/A
Reporting on race, ethnicity, or other socially relevant groupings	N/A
Population characteristics	N/A
Recruitment	N/A
Ethics oversight	N/A

Note that full information on the approval of the study protocol must also be provided in the manuscript.

Field-specific reporting

Please select the one below that is the best fit for your research. If you are not sure, read the appropriate sections before making your selection.

☒ Life sciences ☐ Behavioural & social sciences ☐ Ecological, evolutionary & environmental sciences

For a reference copy of the document with all sections, see nature.com/documents/nr-reporting-summary-flat.pdf

Life sciences study design

All studies must disclose on these points even when the disclosure is negative.

Sample size	The sample size was 3 biological replicates for each condition and experiment.
Data exclusions	No data was excluded.
Replication	The analysis was performed with 3 biological replicates.
Randomization	All samples were measured randomised.
Blinding	Investigators were not blinded to group allocation during data collection and analysis. The study and conclusions are of technical nature, and thus not prone to a potential observer bias.

Reporting for specific materials, systems and methods

We require information from authors about some types of materials, experimental systems and methods used in many studies. Here, indicate whether each material, system or method listed is relevant to your study. If you are not sure if a list item applies to your research, read the appropriate section before selecting a response.

Materials & experimental systems

n/a	Involved in the study	
☐	☒	Antibodies
☒	☐	Eukaryotic cell lines
☒	☐	Palaeontology and archaeology
☐	☒	Animals and other organisms
☒	☐	Clinical data
☒	☐	Dual use research of concern
☒	☐	Plants

Methods

n/a	Involved in the study	
☒	☐	ChIP-seq
☐	☒	Flow cytometry
☒	☐	MRI-based neuroimaging

Antibodies

Antibodies used	The following antibodies were used: CD31 (1:100, #102522, Biolegend), CD45 (1:100, #103132, Biolegend), CD235a (1:100, #349105, Biolegend), CD24 (1:100, #101840, Biolegend), CD71 (1:100, #113806, Biolegend), CD49f (1:200, #313612, Biolegend), CD81 (1:100, #740060, BD Biosciences), SIRPA (1:100, #144008, Biolegend), EPCAM (1:100, #118210, Biolegend).
Validation	*Describe the validation of each primary antibody for the species and application, noting any validation statements on the manufacturer's website, relevant citations, antibody profiles in online databases, or data provided in the manuscript.*

Animals and other research organisms

Policy information about studies involving animals; ARRIVE guidelines recommended for reporting animal research, and Sex and Gender in Research

Laboratory animals	C57BL/6JRj, twelve weeks, Janvier Labs (Saint Berthevin, France)
Wild animals	N/A
Reporting on sex	only male C57BL/6JRj were used.
Field-collected samples	Mice were kept on a 12:12 light-dark cycle and provided with water and food ad libitum.
Ethics oversight	The animal studies complied with the ARRIVE guidelines and were conducted accordance with the EU Directive 2010/63/EU for animal experiments and with regard to specific INSERM guidelines. All the experiments were approved by the Ethical Committee of Paris Cité University and the French Ministry of Higher Education and Research (#16376-2017122210502504).

Note that full information on the approval of the study protocol must also be provided in the manuscript.

Plants

Seed stocks	N/A
Novel plant genotypes	N/A
Authentication	N/A

Flow Cytometry

Plots

Confirm that:

☒ The axis labels state the marker and fluorochrome used (e.g. CD4-FITC).

☒ The axis scales are clearly visible. Include numbers along axes only for bottom left plot of group (a 'group' is an analysis of identical markers).

☒ All plots are contour plots with outliers or pseudocolor plots.

☒ A numerical value for number of cells or percentage (with statistics) is provided.

Methodology

Sample preparation	Islets were dispersed in single cell suspensions using the Neural Tissue Dissociation Kit (#130-092-628, Miltenyi Biotec, Bergisch Gladbach, Germany). Cell surface staining was performed as described17. Briefly, dispersed islet cells were centrifuged and resuspended in FACS medium (HBSS 10% Fetal Calf Serum) with antibodies for 15 min at 4∘C in the dark. The following antibodies were used: CD31 (1:100, #102522, Biolegend), CD45 (1:100, #103132, Biolegend), CD235a (1:100, #349105, Biolegend), CD24 (1:100, #101840, Biolegend), CD71 (1:100, #113806, Biolegend), CD49f (1:200, #313612, Biolegend), CD81 (1:100, #740060, BD Biosciences), SIRPA (1:100, #144008, Biolegend), EPCAM (1:100, #118210, Biolegend). Then, cells were rinsed and resuspended in FACS medium (HBSS 10% Fetal Calf Serum) with propidium iodide (1/4,000, #P4864, Sigma Aldrich). For each antibody, the optimal dilution was determined by titration.
Instrument	Cell sorting was carried using a FACS Aria III (BD Biosciences).

Software	Data were analysed using FlowJo™ Software 10.6.1(RRID:SCR_008520, BD Bioscience).
Cell population abundance	post-sort fractions were not acquired because we were limited in sample amount. The purification was accessed by mass spectrometer acquisitions while we showed many quality control markers for high quality of the cell types and FACS populations.
Gating strategy	cells were gated for single cells by FSC-A/SSC-A and FSC-W/FSC-H, for live cells by negative PerCP signal and endocrine cells by positive Epcam signal. Delta cells were sorted from the CD24 high signal population, while alpha were sorted from CD49f and CD71 negative from the CD24 low fraction while beta cells were CD71 and CD49f positive.

☒ Tick this box to confirm that a figure exemplifying the gating strategy is provided in the Supplementary Information.
